# A Wheat *TaTOE1-B1* Transcript *TaTOE1-B1-3* Can Delay the Flowering Time of Transgenic *Arabidopsis*

**DOI:** 10.3390/ijms222312645

**Published:** 2021-11-23

**Authors:** Tianqi Song, Yang Yu, Mingfei Zhang, Hongwei Zhou, Shuangxing Zhang, Ming Yu, Jianfei Zhou, Jie Cheng, Jishan Xiang, Songjie Yang, Xiaoke Zhang

**Affiliations:** 1 College of Agronomy, Northwest A&F University, Yangling 712100, China; songtiq@163.com (T.S.); yuyang0319@nwafu.edu.cn (Y.Y.); zhouhw12356@163.com (H.Z.); zsx18211672769@163.com (S.Z.); nwafuyuming@163.com (M.Y.); zhoujianfei369@163.com (J.Z.); chengjie9322@163.com (J.C.); 2 Academy of Agricultural Sciences, Key Laboratory of Agro-Ecological Protection & Exploitation and Utilization of Animal and Plant Resources in Eastern Inner Mongolia, Chifeng University, Chifeng 024000, China; zhangmingfei0207@163.com; 3 School of Modern Agriculture & Biotechnology, Ankang University, Ankang 725000, China; akxyysj@163.com

**Keywords:** wheat, flowering time, *TaTOE1-B1*, *TaTOE1-B1-3*

## Abstract

Flowering time is one of the most important agronomic traits in wheat production. A proper flowering time might contribute to the reduction or avoidance of biotic and abiotic stresses, adjust plant architecture, and affect the yield and quality of grain. In this study, *TaTOE1-B1* in wheat produced three transcripts (*TaTOE1-B1-1*, *TaTOE1-B1-2*, and *TaTOE1-B1-3*) by alternative splicing. Compared to the longest transcript, *TaTOE1-B1-1*, *TaTOE1-B1-3* has a deletion in the sixth exon (1219–1264 bp). Under long-day conditions, the heterologous overexpression of the *TaTOE1-B1-3* gene delayed flowering, prolonged the vegetative growth time, and enlarged the vegetative body of *Arabidopsis*, but that of *TaTOE1-B1-1* did not. As typical AP2 family members, TaTOE1-B1-1 and TaTOE1-B1-3 are mainly located in the nucleus and have transcriptional activation activities; the transcriptional activation region of TaTOE1-B1-3 is located in the C-terminal. In *TaTOE1-B1-3* overexpression lines, the expression of flowering-related *AtFT* and *AtSOC1* genes is significantly downregulated. In addition, this study confirms the protein–protein interaction between TaTOE1-B1-3 and TaPIFI, which may play an important role in flowering inhibition. These results provide a theoretical basis for the precise regulation of wheat flowering time.

## 1. Introduction

During the long course of evolution, plants have developed powerful adaptive mechanisms to cope with a variety of environments [1,2]. Flowering is an important stage in the process of plant life generation. When plants grow to a certain stage, under the combined action of various internal and external factors, a series of metabolic pathways are activated to initiate the process of flower development in order to carry out reproductive growth [3]. According to studies in model plant *Arabidopsis* (*Arabidopsis thaliana*), flowering in higher plants is mainly regulated by metabolic pathways, such as the photoperiod pathway, in response to day length and light quality; the vernalization pathway, sensitive to temperature; the GA pathway, which regulates reproductive development by endogenous hormones; and the autonomic pathway, which depends on its own development status [4,5,6]. An appropriate flowering time is an important guarantee for seed plants to complete life generation and maintain species reproduction. Meanwhile, in long-term crop cultivation, domestication, and agricultural production practice, flowering time is among the agronomic traits we focus on.

Wheat (*Triticum aestivum* L.) is one of the most important food crops worldwide. The strong environmental adaptability of wheat is an important reason for its widespread cultivation [7,8,9]. In wheat production, appropriate flowering is helpful in coping with adverse environmental factors such as heat, cold, freeze, heavy rainfall, and harmattan, as well as various fungal diseases such as powdery mildew, scabbed mildew, and rust [10]. At the same time, it is also conducive to obtaining a high yield and better quality, by the coordination of vegetative and reproductive growth [11]. Therefore, it is of considerable significance to explore and identify important flowering-related genes in wheat, understand the molecular mechanism of wheat flowering regulation, and realize precise regulation of flowering time in wheat breeding in order to improve grain yield and ensure food security.

As wheat is a typical higher plant that is sensitive to temperature and light, its flowering is mainly regulated by the vernalization and photoperiod pathways. At the early developmental stages, a certain intensity of low temperature determines whether winter wheat will change from vegetative to reproductive growth normally [12]. Four groups of vernalization genes (*Vrn*) are mainly involved in the formation of temperature-sensing characteristics of wheat, and diverse allelic variations and their various vernalization gene combinations determine the requirements of different strains of wheat based on temperature [13,14,15,16]. The photoperiod genes (*Ppd*) are considered to be mainly involved in the circadian rhythm response in wheat, and variations in *Ppd* genes are the main reason for the differences in sensitivity to day length in wheat [4,17,18]. In addition, flowering regulation in higher plants often involves complex regulatory mechanisms at multiple levels. With the rapid development of molecular biology and bioinformatics research, a series of transcription factors related to flowering have been reported, deepening our understanding of plant flowering regulation [19].

The AP2/ERF superfamily is one of the most numerous transcription factor families in higher plants [20]. According to its molecular and structural characteristics, the AP2/ERF superfamily is generally divided into five subfamilies: APETALA2 (AP2), ethylene-responsive factor (ERF), related to ABI3/VP1 (RAV), dehydration-responsive element binding (DREB) protein, and soloist [21]. Previous studies generally showed that some members of the AP2 subfamily are closely related to plant development. The *Q* gene, a widely studied member of the AP2 subfamily, is believed to play an important role in controlling wheat spike shape development and shattering behavior [22,23,24,25]. In recent years, with the deepening of research, it has been reported that AP2 subfamily members are involved in floral organ development, flowering time regulation, and seed size and shape control [26,27,28].

Two AP/ERF superfamily members in maize (*Zea mays* L.), *Glossy15* and *ZmTOE1*, have been reported as inhibitors of flowering [29,30]. Wheat *TaTOE1-B1* has been reported as a potential floral inhibitor, and the expression level of *TaTOE1-B1* gene significantly decreases from the third to the fourth week and transfer from the short-day to long-day in some wheat cultivars [31]. In *Arabidopsis*, there are three *TOE* genes, *AtTOE1*, *AtTOE2*, and *AtTOE3* [19]. AtTOE1 negatively regulates *FLOWERING LOCUS T* (*FT*), independent of CONSTANS (CO), which is an integrator of photoperiodic signals, and leads to delayed flowering [32]. AtTOE1 also affects the stability of CO in both direct and indirect ways: upstream of *FT*, decreased CO stability directly downregulates the expression of *FT*, thus inhibiting flowering [19]. The interaction between AtTOE3 and AGAMOUS (AG) in *Arabidopsis* plays an important role in floral organ morphogenesis, and post-transcriptional regulation by miRNA172 on *TOE* gene is vital to flowering and floral organ development in plants [33]. These results suggest that some members of the AP2 family in different species have the function of regulating plant flowering.

At present, there are still few reports on *TOE* genes in wheat. Although the gene locus of *TaTOE1-B1* is considered a floral suppression site, which was previously identified by using doubled haploid population of Avalon × Cadenza crosse, we also lack intuitive phenotypic evidence, and the molecular mechanism of *TaTOE1* gene involved in the regulation of flowering time is still not clear [31]. In addition, it was also discovered that the *TaTOE1-B1* genome produces multiple transcripts through alternative splicing. The three transcripts may have different biological functions, and which transcript plays the primary role in flowering inhibition is still unclear. This study further confirms that *TaTOE1-B1-3* may be the key transcript in delaying flowering time and preliminarily explores its molecular mechanism. This work further enriches our understanding of *TOE* genes in plants and provides a reference for understanding the adaptive formation mechanism of wheat. At the same time, it also provides a theoretical basis for the regulation of flowering time in the practice of wheat breeding.

## 2. Results

### 2.1. Bioinformatics Analysis of TaTOE-B1

**Figure 1:**
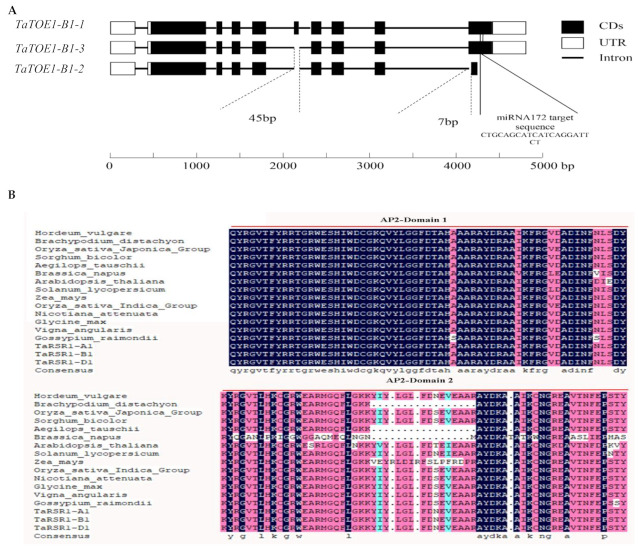
Gene structure analysis and protein sequence alignment. (**A**) Gene structure analysis of *TaTOE1-B1-1*, *TaTOE1-B1-2*, and *TaTOE1-B1-3*. Dotted line shows relative locations where differences between transcripts appear; solid line shows relative locations of miRNA172 target; (**B**) AP2 domain sequence alignments of TaTOE1 homologous proteins in different species.

Based on the annotation analysis, the wheat TaTOE1-B1 gene can generate three alternative splice variants: TaTOE1-B1-1 (ID: TraesCS1B02G076300.1), TaTOE-B1-2 (ID: TraesCS1B02G076300.2), and TaTOE1-B1-3 (ID: TraesCS1B02G076300.3) [31,34]. TaTOE1-B1-1 is the longest transcript, with a length of 2227 bp. Compared to TaTOE1-B1-1, TaTOE-B1-2 and TaTOE1-B1-3 have a deletion of 45 bp (1219–1264 bp) in the coding sequence (CDS). The deletion site (1219–1264 bp) is located in the second AP2 domain, and the loss of the 45 bp fragment does not cause a frameshift mutation. In addition to the above deletion, TaTOE-B1-2 has another 7 bp (1603–1611 bp) deletion in the CDS, which can lead to frameshift mutation and the premature termination of translation (Figure 1A and Figure S1). TaTOE1-B1-1 encodes a protein of 512 amino acids (Figure S2), with a molecular weight (MW) of about 55.7 kDa and a theoretical isoelectric point (PI) of 6.47; TaTOE1-B1-3 encodes a protein of 497 amino acids, with a MW of about 54.0 kDa and a theoretical PI of 6.71, and TaTOE-B1-2 encodes a protein of 434 amino acids, with a MW of about 47.5 kDa and a theoretical PI of 6.33. The conserve domain identification shows that all three transcripts have two AP2 domains (AP2-domain 1 and AP2-domain 2) and belong to the AP2 family.

**Figure 2:**
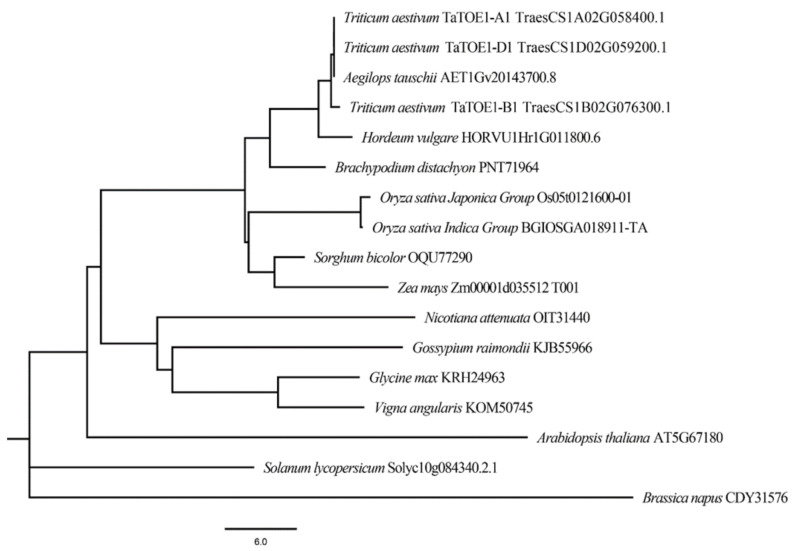
Phylogenetic tree constructed by neighbor-joining (NJ) method (bootstrap = 1000). Species name and protein ID of homologous proteins shown at ends of branches.

Next, the longest protein, TaTOE1-B1-1, was used as the input sequence to retrieve homologous proteins, and proteins highly homologous to wheat TaTOE1-B1-1 were retrieved in various species. Sequence alignment analysis shows that the homologous proteins in different species are highly conserved, and the first AP2 domain (AP2-domain 1) is more conserved than the second (AP2-domain 2) (Figure 1B). Phylogenetic analysis based on these proteins shows that TaTOE1-B1-1 homologous proteins in monocot and dicot plants are clearly divided into two sub-branches, and the degree of homology gradually increases with the recursion of genetic relationship among species. TaTOE1-B1-1 is closely related to RSR1, which has been reported in rice, and *Arabidopsis* TOE3 is a direct homologous protein of TaTOE1-B1-1 in the model plant *Arabidopsis* (Figure 2). In addition, it is worth noting that proteins with a 15 amino acid deletion similar to TaTOE1-B1-3 are also found in some species. These results suggest that the alternative splicing reported in this study may be prevalent in multiple species.

### 2.2. Cloning of TaTOE1-B1-1 and TaTOE1-B1-3

Due to the obvious premature transcription termination of *TaTOE-B1-2*, we tried to isolate the full-length CDS of *TaTOE1-B1-1* and *TaTOE1-B1-3* from the total cDNA of common wheat cultivar Chinese Spring by RT-PCR with a pair of common primers (Clone-F and Clone-R) (Table S1). Sequencing results show that the obtained sequences were consistent with the database description, indicating that we successfully obtained the complete CDS of *TaTOE1-B1-1* and *TaTOE1-B1-3*. In addition, the abundance of the two transcripts may vary in different tissues, and the *TaTOE1-B1-1* sequence is more easily cloned in root tissue than in leaf. Among the nine positive clones used for sequencing, most of them were *TaTOE1-B1-1* and only two were *TaTOE1-B1-3*. These results also indicate that the two transcripts may have some spatial and temporal expression characteristics.

### 2.3. Overexpression of TaTOE1-B1-3 Results in Considerable Delayed Flowering Time in Transgenic Arabidopsis

**Figure 3:**
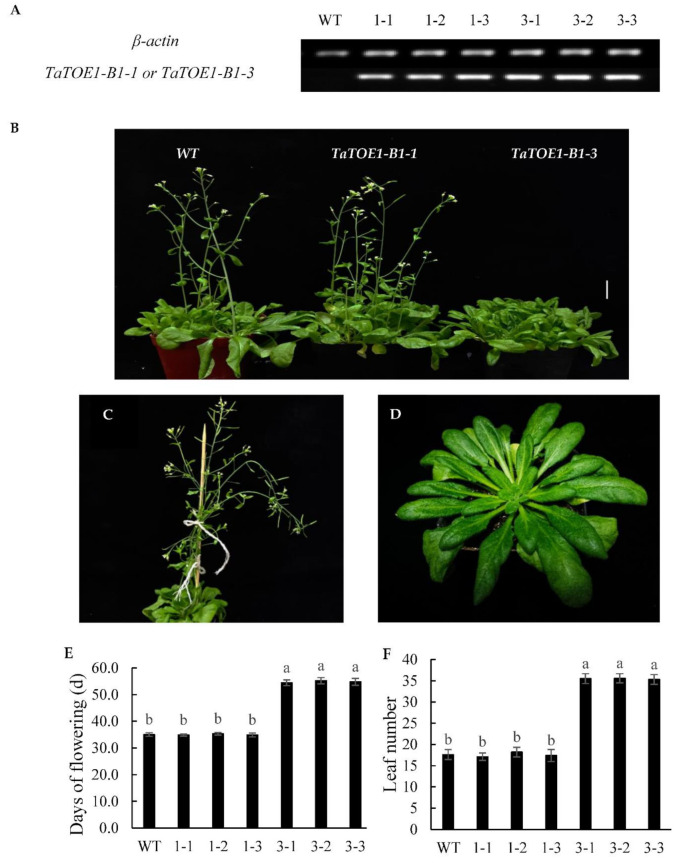
Phenotypic identification of *Arabidopsis* transgenic lines. (**A**) Gene expression analysis of *TaTOE1-B1-1* and *TaTOE1-B1-3*. WT, wild-type; 1-1, 1-2, 1-3: three lines of transgenic *TaTOE1-B1-1*; 3-1, 3-2, 3-3: three lines of transgenic *TaTOE1-B1-3*; (**B**) Phenotype of *TaTOE1-B1-1* and *TaTOE1-B1-3* transgenic lines under long-day conditions (bar = 2 cm); (**C**) WT line at 49 days; (**D**) *TaTOE1-B1-3* transgenic line at 49 days; (**E**) Days of flowering; (**F**) Number of rosette leaves. Data presented in (**E**,**F**) are means ± SD of each line (*n* > 10). Different lowercase letters above bars indicate significant difference at *p* < 0.05.

*TaTOE1* genes (*TaTOE1-A1*, *TaTOE1-B1*, and *TaTOE1-D1*) exist in three wheat sub-genomes simultaneously, and each one can generate multiple transcripts, which makes it more difficult to analyze the functions of different transcripts. In order to conduct such an analysis, overexpressed *Arabidopsis* transgenic lines of *TaTOE1-B1-1* and *TaTOE1-B1-3* were constructed. PCR and SqRT-PCR identification of transgenic *Arabidopsis* indicate that the target genes integrated into the *Arabidopsis* genome were efficiently expressed in the heterologous system (Figure 3A). The transgenic progeny of *TaTOE1-B1-1* and *TaTOE1-B1-3* show different phenotypic characteristics (Figure 3B–D). Under long-day (16 h light/8 h dark) conditions, the wild-type (WT) and *TaTOE1-B1-1* transgenic lines began flowering at about 35 days and had 17 rosette leaves (Figure 3E,F). There was no significant difference between the WT and *TaTOE1-B1-1* transgenic lines. *TaTOE1-B1-3* lines began flowering at about 55 days and had 36 rosette leaves, which is significantly different from the WT and *TaTOE1-B1-1* transgenic lines. These results show that heterologous overexpression of *TaTOE1-B1-3* leads to a serious delay in flowering time, and a significantly higher number of rosette leaves than in WT and *TaTOE1-B1-1* transgenic lines.

### 2.4. Overexpression of TaTOE1-B1-3 Downregulates AtFT and AtSOC1 Expression in Transgenic Arabidopsis

In *Arabidopsis*, heterologous expression of wheat *TaTOE1-B1-3* significantly delays flowering. In order to further elucidate the mechanism of *TaTOE1-B1-3*, we investigated its effects on several key flowering genes in *Arabidopsis*. *AtFT*, a direct flowering promotion factor integrating photoperiodic signal is regulated by *AtCO*; *AtSOC1*, an integrator of vernalization and autonomic pathways, is regulated by *AtFLC* and directly promotes flowering [6,35].

**Figure 4:**
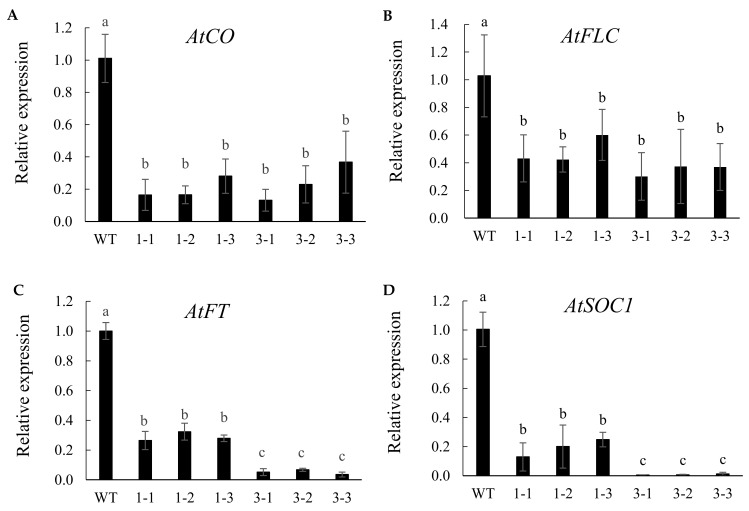
Quantitative analysis of flowering-related genes in *Arabidopsis* when WT is flowering: (**A**) *AtCO*; (**B**) *AtFLC*; (**C**) *AtFT*; (**D**) *AtSOC1*. WT, wild-type; 1-1, 1-2, 1-3: three lines of transgenic *TaTOE1-B1-1*; 3-1, 3-2, 3-3: three lines of transgenic *TaTOE1-B1-3*. Each value represents a plant line containing at least three biological and three technical replicates. Data presented are means ± SD. Different lowercase letters above bars indicate significant difference at *p* < 0.05.

Compared to WT, overexpression of *TaTOE1-B1-3* or *TaTOE1-B1-1* significantly downregulates the expression of *AtCO*, *AtFT*, *AtFLC*, and *AtSOC1* (Figure 4). Notably, although *AtCO* and *AtFLC* expression is reduced, there are no significant differences between *TaTOE1-B1-3* and *TaTOE1-B1-1* transgenic lines (Figure 4A,B). The inhibition of *AtFT* and *AtSOC1* expression in *TaTOE1-B1-3* transgenic lines is significantly stronger than in *TaTOE1-B1-1* (Figure 4C,D).

### 2.5. TaTOE1-B1-1 and TaTOE1-B1-3 Are Localized in the Nucleus and Have Transcriptional Activation Activity

**Figure 5:**
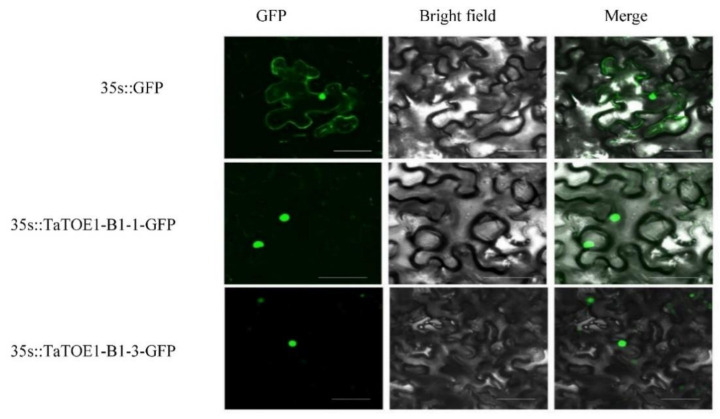
Subcellular localization of TaTOE1-B1-1 and TaTOE1-B1-3 in tobacco leaves (bar = 50 μm).

Subcellular localization can help us to understand the biological function of the protein. Under confocal microscope, the fluorescence signals are concentrated in the nucleus, indicating that TaTOE1-B1-1 and TaTOE1-B1-3 are mainly located in the nucleus (Figure 5).

**Figure 6:**
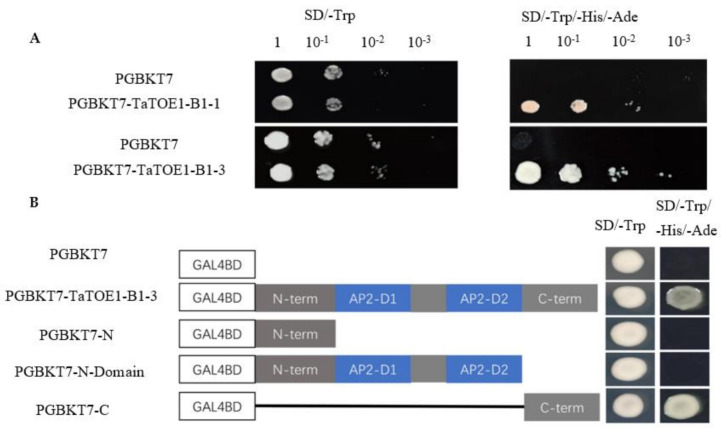
(**A**) Transcriptional activation activity analysis of TaTOE1-B1-1 and TaTOE1-B1-3; (**B**) Location analysis of transcriptional activation region of TaTOE1-B1-3.

Detection of yeast toxicity and transcriptional activation provides the basis for further exploration of the mechanism of TaTOE1-B1-3 in the eukaryotic model system. TaTOE1-B1-1 and TaTOE1-B1-3 have no significant toxic effects on the yeast system, and both have transcriptional activation activity, which confirms that these two proteins may be involved in the transcriptional regulation of gene expression as transcription factors in the nucleus (Figure 6A). The transcriptional activation domain is found to be located in the C-terminal of TaTOE1-B1-3 (Figure 6B).

### 2.6. Identification of Interaction between Proteins TaTOE1-B1-3 and TaPIFI

**Figure 7:**
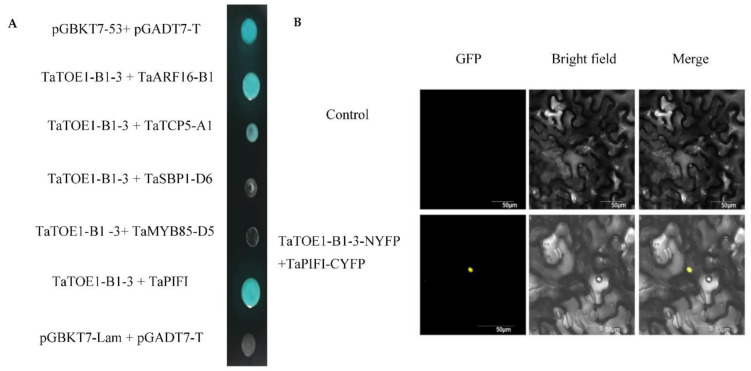
Supplementary validation of TaTOE1-B1-3 interacting with candidate proteins. (**A**) Yeast point-to-point detection; (**B**) BiFC detection (bar = 50 μm).

In order to further clarify the molecular mechanism of TaTOE1-B1-3, we screened its interaction proteins. First, through an online STRING database prediction, we identified several candidate proteins that may interact with TaTOE1-B1-3, and finally isolated four interacting protein coding genes from wheat named *TaTCP5-A1*, *TaARF16-B1*, *TaSBP1-D6*, and *TaMYB85-D5*. Point-to-point analysis in the Y2H system shows that TaTOE1-B1-3 can interact with TaARF16-B1 and TaTCP5-A1, but the fluorescent signals are almost invisible in the BiFC system. This may indicate that the interaction is either too weak or nonexistent (Figure 7A). Then, the interaction proteins of TaTOE1-B1-3 were further screened through mating, and some positive clones were screened. According to the database annotation information, we finally selected a candidate protein, TaPIFI, for further verification; the interaction was verified in the yeast system, and the interaction signal in the BiFC system was mainly concentrated in the nucleus (Figure 7A,B).

## 3. Discussion

Flowering time is a key stage in the life generation of seed plants and a proper flowering time is crucial for plants. In wheat production, flowering time is also one of the prime agronomic traits that breeders focus on. Understanding the molecular mechanisms of plant adaptation, identifying the key genes involved in crop adaptation, and exploiting the available genetic resources in major crops are important for us to cope with climate change and improve grain yield. This study further confirms that *TaTOE1-B1-3* may be the functional transcript of *TaTOE1-B1* in wheat flowering time regulation. Under long-day conditions, overexpression of *TaTOE1-B1-3* results in prolonged vegetative growth time, delayed flowering time, and enlarged vegetative body. TaTOE1-B1-3, as a transcription factor, delays flowering by inhibiting the expression of key flowering genes, *FT* and *SOC1*, and the interaction relationship between TaPIF1 and TaTOE1-B1-3 is identified, which deepens our understanding of *TOE* genes in plants. At the same time, it also provides available resources for the genetically engineered breeding of some plants that use plant nutrients as harvesting organs, such as forage grass and vegetables [36].

### 3.1. Differences in 15 Amino Acids May Be Important to the Functional Difference between TaTOE1-B1-1 and TaTOE1-B1-3

The existence of alternative splicing in plants shows the specialization of gene function in the process of biological evolution, which is considered a universal phenomenon in organisms. The expression characteristics of different transcripts produced by the same gene, the functional positions of proteins coding by these transcripts, and the biological functions of these proteins may be different [37,38]. Previous studies have shown that *MaMYB16* produces two transcripts, *MaMYB16L* and *MaMYB16S*, with opposite functions in controlling fruit ripening through alternative splicing [39]. MaMYB16L is a transcription inhibitor that inhibits fruit ripening by directly downregulating the expression of *MaDREB2*; MaMYB16S loses the ability to bind to DNA, but can bind to MaMYB16L competitively, and then form inactive dimers that ultimately promote fruit ripeness.

In this study, three transcripts (*TaTOE1-B1-1*, *TaTOE1-B1-2*, and *TaTOE1-B1-3*) were generated from the same genome DNA by alternative splicing. Our experiments prove that heterologous overexpression of *TaTOE1-B1-3* reveals a significant delay in flowering time of transgenic *Arabidopsis*, but that of *TaTOE1-B1-1* did not. Referring to relevant reports, we preliminarily speculate that there may be two main reasons for this difference. First, the loss of the sixth exon results in a difference of 15 amino acids within the second AP2 domain of TaTOE1-B1-1 and TaTOE1-B1-3, which might be the key to the phenotypic difference. Second, some previous studies showed that miRNA172 plays an important role in the regulation of AP2 family members in flower organ differentiation in plants [24,32,33,40,41]. Overexpression of *TaTOE1-B1-1* had no effect on flowering date. This suggests that the potential regulatory mechanisms in this study might not be similar to the reported regulatory mechanism of MaMYB16. Whether amino acid deletion leads to TaTOE1-B1-3 recognition of specific downstream genes has not been confirmed. Although the miRNA172 target sequences of the two transcripts are completely identical, we questioned whether the flanking sequence would affect the mRNA secondary structure and further influence the target recognition of miRNA172. We used a semi-quantitative method to preliminarily identify the expression of two transcripts in transgenic *Arabidopsis*, and the results show that both transcripts are effectively expressed (Figure 3A). This suggests that the phenotypic differences may not be caused by miRNA172-mediated post-transcriptional regulation. We are inclined to think that the differences in these 15 amino acids may be vital to the functional differences between the two transcripts, which needs to be further proven. If the hypothesis is correct, the following work will verify whether *TaTOE-B1-2* has the function of delaying flowering due to the absence of the sixth exon.

### 3.2. The Main Reason for Flowering Delay May Be Downregulated Expression of FT and SOC1

As a floral inhibitor, TaTOE1-B1-3 has effects on some key genes in the floral pathway of plants. There are many reports on downstream genes regulated by TOEs in plants, but they are not systematic. Many studies have revealed that TOEs regulate *CO*, while others have confirmed that TOEs directly regulate *FT* [19]. *TOE* genes are thought to be involved in the photoperiod pathway; however, whether they influence key factors in the vernalization and autonomous pathways has not been reported in detail.

In order to further understand the mechanism of TaTOE1-B1-3, we investigated its effects on the expression of several flower-forming key genes in model plants using heterogenous transgenic lines [3,6]. The results in this study reveal that overexpression of both *TaTOE1-B1-1* and *TaTOE1-B1-3* could inhibit the expression of *AtCO* and *AtFLC*, but there was no significant difference between the transgenic lines. Overexpression of *TaTOE1-B1-3* showed stronger inhibition of *AtFT* and *AtSOC1*, which was partially consistent with some previous studies [42,43,44]. Therefore, inhibition of *AtFT* and *AtSOC1* expression by overexpression of *TaTOE1-B1-3* is important for delayed flowering, but whether this inhibition is direct or indirect is still unclear. We further attempted to analyze the promoter regions of *FT* and *SOC1* in *Arabidopsis* and wheat, and found a large number of *cis*-elements recognized by AP2/ERF transcription factors, such as WBOXNTERF3 and RAV1AAT. This may also imply that TaTOE1-B1-3 regulates the expression of *FT* and *SOC1* directly.

*AtFT* and *AtSOC1* are direct determinants of flower formation in plants [35]. In this study, a noteworthy problem is that, although the overexpression of *TaTOE1-B1-1* had a certain inhibitory effect on the expression levels of *AtCO*, *AtFLC*, *AtFT*, and *AtSOC1* in *Arabidopsis*, it was consistent with the flowering time of WT. It is interesting that the expression level of *AtFT* and *AtSOC1* underwent stronger inhibition in *TaTOE1-B1-3* transgenic lines, and the flowering time was seriously delayed. Previous studies have shown that the expression of *Ppd* genes changes with day length. The expression of *Ppd* genes reached a peak when the day length reached a threshold. Similar characteristics have been reported in some studies on *CO* [4,19]. Therefore, we preliminarily speculate that *FT* and *SOC1* in plants may be directly or indirectly affected by the external environment and internal factors, and thus have a dose-dependent mode of action. However, this study only focuses on the functional differences of three transcripts of *TaTOE1-B1*. As for their expression characteristics, we have not been able to speculate at present.

### 3.3. TaTOE1-B1-3 May Act as a Broad Floral Inhibitor in Plants

The *AtTOE1* gene is seen as a major floral inhibitor and has a high expression level in the early stage of *Arabidopsis*. With the gradual decrease of the expression level of *AtTOE1* gene, the inhibitory effect on floral formation is gradually weakened [19]. Under the action of environmental signals and internal factors, *AtTOE1* expression is gradually decreased in a variety of direct and indirect ways, such as changing its initial expression level and the post-transcriptional regulation mechanism mediated by miRNA172, so that plants can start flower development at an appropriate time [19,32]. In wheat, previous studies detected similar changes in the expression level of *TaTOE1-B1-3* gene during the critical period of vegetative growth to reproductive growth, which preliminarily indicates that this gene in wheat may have a function similar to *AtTOE1* in *Arabidopsis* [31]. In addition, qRT-PCR results show that TaTOE1-B1-3 inhibits *AtFLC* and *AtSOC1* to a certain degree. *AtFLC* is considered as the signal integration factor of vernalization and autonomous pathways. These results suggest that the TOEs may act as universal flower suppressors in plants, not just in the photoperiodic pathway. However, the cDNA library screening in this study still obtained multiple chloroplast targeted photosystem proteins, indicating that TaTOE1-B1-3 may mainly affect flowering time through the photoperiod pathway.

### 3.4. Protein Interaction between TaTOE1-B1-3 and TaPIFI May Play an Important Role in Flowering Inhibition

Flowering is observed as an external expression, but it involves multiple physiological and biochemical processes inside plants. In order to further understand the molecular mechanism of TaTOE1-B1-3, we tried to screen its interacting proteins. Verification of the candidate interaction proteins shows that TaPIFI has a strong interaction effect with TaTOE1-B1-3. In *Arabidopsis*, PIFI is found to be encoded by a nuclear gene and participates in photoinhibition, chlororespiration, and heat stress, which may play an important role in NAD(P)H dehydrogenase complex-mediated chlororespiratory electron transport [45]. There are also some reports showing that photosynthetic components located in chloroplasts may affect the expression of nuclear genes, which corroborates our results [46]. Therefore, we speculate that the interaction between TaTOE1-B1-3 and TaPIFI may play a certain role in regulating the expression of some flowering-related genes, which is the subject of future work.

## 4. Materials and Methods

### 4.1. Plant Materials and Growing Conditions

The wheat cultivar, Chinese Spring, was used to clone the different transcripts of *TaTOE1-B1*. Wheat seedlings were cultured in a light incubator at temperatures of 25/19 °C (day/night) and 16/8 h light/dark cycle and irrigated with 1/2 MS nutrient solution. After being cultured for 3–4 weeks, fresh samples of different tissues were taken and total RNA of wheat was extracted.

*Arabidopsis* (ecotype Columbia) was used to construct transgenic lines of target genes. After sterilization, the seeds were laid on solid MS medium. Two weeks after germination, the seeds were transplanted to culture soil and cultivation was continued in the light incubator (16/8 h light/dark) until further use.

*Nicotiana benthamiana* plants were used for subcellular localization and bi-molecular fluorescence complementarity. The seeds were first spread in culture soil and cultured in a light incubator (16/8 h light/dark). After 2 weeks, we transplanted them to small pots and continued to culture them for about 3–4 weeks.

### 4.2. Bioinformatics Analysis of Sequence

The genome DNA, cDNA, CDS, and protein sequence of target genes were downloaded from the EnsemblPlants database (http://plants.ensembl.org/index.html, accessed on 17 September 2021), and information on the 3 transcripts product by the *TaTOE1-B1* genome was obtained and used to analyze the gene structure. The ProtParam online tool (https://web.expasy.org/protparam/, accessed on 17 September 2021) was used to predict the physical and chemical properties of proteins that target gene encoding. The CDD (https://www.ncbi.nlm.nih.gov/Structure/cdd/wrpsb.cgi, accessed on 17 September 2021) was used to identify the conserved domains contained in target proteins. The EnsemblPlants online BLAST tool was used to obtain the protein sequences of the homologous *TaTOE-B1-1* genes in multiple species, and the longest protein sequence encoded by *TaTOE1-B1-1* was used as the input sequence. DNAMAN V9 was used for multi-sequence alignment. The phylogenetic tree was constructed by using MEGA7.0, the neighbor-joining (NJ) method and bootstrap set to 1000 times. *Cis*-element analysis was performed by using the new PLACE online tool (https://www.dna.affrc.go.jp/PLACE/?action=newplace, accessed on 17 September 2021).

### 4.3. Isolation of TaTOE-B1-1 and TaTOE1-B1-3

Due to the high degree of sequence consistency between different transcripts, according to the reference sequence of wheat, a pair of common primers (Clone-F and Clone-R) were designed for the cloning of *TaTOE-B1-1* and *TaTOE1-B1-3* (Table S1), and the different transcripts were identified by sequencing. Wheat total RNA was extracted by using the Trizol method. After the RNA was detected by electrophoresis and confirmed to be available, total cDNA was obtained by reverse transcription using EasyScript^®^ One-Step GDNA Removal and cDNA Synthesis Supermix (TransGen, Beijing, China). Wheat total cDNA was used as a template, and Phanta Max Super-Fidelity DNA Polymerase (Vazyme, Nanjing, China) was used to amplify the CDS of the target gene by PCR.

### 4.4. Construction of TaTOE1-B1-1 and TaTOE1-B1-3 Transgenic Arabidopsis Lines

Homologous arm primers with 5′-flanking restriction endonuclease sites were designed to amplify the target genes. Plant overexpression vector pBI121 was used to construct the recombinant overexpression vectors. After digestion by the restriction endonuclease *BamH*I and *Sac*I (New England Biolabs, Ipswich, MA, USA), the plasmid was reconstructed by a ClonExpress^®^ II one-step cloning kit (Vazyme, Nanjing, China) to obtain the overexpression vectors of *TaTOE1-B1-1* and *TaTOE1-B1-3*. *Arabidopsis* inflorescence was infected using a dipping method to obtain transgenic lines [47]. The transgenic progeny was screened on solid MS medium containing 50 μg/mL kanamycin (Kan). The screened positive seedlings were transplanted into culture soil, and then the DNA of plant leaves was extracted at the sixth week for genome identification. The total PCR volume (20 µL) was composed of 10 µL of 2× PCR mix, 1 µL of DNA template, 1 µL of forward primer (pBI-CDs-F), 1 µL reverse primer (pBI-CDs-R), and 7 µL of sterile nuclease-free water, and the conditions for the PCR were as follows: 95 °C for 3 min, 35 cycles of 95 °C for 30 s, 70 °C for 30 s, 72 °C for 1 min, and 72 °C for 5 min.

### 4.5. Phenotypic Identification of Transgenic Progeny

The transgenic positive lines were propagated to T_3_ generation for phenotypic identification. WT *Arabidopsis* was used as control to evaluate the phenotypes of *TaTOE1-B1-1* and *TaTOE1-B1-3* transgenic progeny. First, the primer pair Clone-F and Clone-R was used to test the target gene expression level in *Arabidopsis* progeny by semi-quantitative reverse transcription and polymerase chain reaction (SqRT-PCR). Then, the number of leaves in the rosette were counted when the plants produced the first flower, the days from germination to flowering of each line were counted, and some pictures were taken [35].

### 4.6. qRT-PCR Analysis

Leaves of transgenic lines and WT were obtained when the WT *Arabidopsis* began flowering. To eliminate the effects of the circadian clock on flowering-related genes, all plants were grown under long-day conditions and samples were taken 3 h after dawn. Each transgenic sample contained 3 lines, and RNA extraction and reverse transcription were performed as described above. *AtPP2A* was used as an internal reference, the expression of several flowering-related genes (*AtCO*, *AtFT*, *AtSOC1*, and *AtFLC*) was analyzed by using PerfectStart^TM^ Green qPCR Supermix (Transgen, Beijing, China), the primer sequence and reaction procedures referred to Wang’s method, and the expression level was evaluated using the 2^−ΔΔCt^ method [35,48]. Each sample contained at least 3 biological and 3 technical replicates.

### 4.7. Subcellular Localization

The open reading frames of *TaTOE1-B1-1* and *TaTOE1-B1-3*, excluding the stop codon (TGA), were amplified and subcloned into the pCAMBIA1302-GFP vector (*CaMV35*s promoter), resulting in *CaMV35*s::TaTOE1-B1-1-GFP and *CaMV35*s::TaTOE1-B1-3-GFP. The corresponding primers are listed in Table S1. GFP driven by the *CaMV35*s promoter (*CaMV35*s::GFP) was used as control. After the recombinant vector was sequenced correctly, it was transformed into Agrobacterium GV3101 strain and propagated in LB liquid medium containing 50 μg/mL Kan, 25 μg/mL rifampin (Rif), and 50 μg/mL gentamicin (Gen). Resuspended buffer solution (100 mM/mL magnesium chloride (MgCl_2_), 100 mM/mL 2-(*N*-morpholine) sulfonic acid (MES), 200 μm/mL acetosyringone (AS)) was used for transient expression in tobacco leaves. The fluorescence signal was observed by FV-1200 laser confocal microscope (Olympus, Tokyo, Japan) under excitation of 488 nm [49].

### 4.8. Transcriptional Activator Activity Analysis

Two restriction endonuclease sites, *EcoR*I and *Sal*I, were selected, and the coding sequences of *TaTOE1-B1-1* and *TaTOE1-B1-3* were inserted into the bait vector pGBKT7 by homologous recombination. The recombinant vector was transferred into Y2HGold strain yeast and coated on SD/-Trp solid medium. Mono-clones were selected and cultured at a constant temperature and shaken for 3–5 days (30 °C, 250 rpm). The yeast solution was adjusted to OD_600_ = 1.0, diluted at a gradient of 1, 10^−1^, 10^−2^, and 10^−3^, then divided into 1 μL portions by dropping it on SD/-Trp and SD/-Trp/-His/-Ade solid medium. The growth of the colonies was observed and recorded after 3–5 days of inverted culture at 30 °C to determine whether there was self-activation and toxicity according to colony status.

### 4.9. Prediction and Library Screening of Candidate TaTOE1-B1-3 Interaction Proteins

The STRING protein interaction database (https://string-db.org/cgi/input.pl, accessed on 17 September 2021) was used to predict the protein interaction network of TaTOE1-B1-3, then the prediction proteins were used as input sequences to search the wheat genome protein database in a species-limited way, and the candidate proteins in wheat were identified. According to the annotated information of these candidate proteins, 4 candidate proteins that may be related to flowering were screened and the coding regions of these genes were isolated from wheat cDNA by using RT-RCR.

According to the results of transcriptional activation activity, the bait vector (pGBKT7-N-domain) was used to screen interacting proteins in a wheat cDNA library, and the candidate proteins were screened according to the process of the yeast bimating screening library in the nuclear system (Oebiotech, Shanghai, China). The wheat cDNA library used in this experiment was constructed by Wei [50]. The candidate clones were stained with X-α-gal on SD/-Trp/-His-/Leu/-Ade medium. Monoclonals that could change to blue were selected and their plasmid was extracted, then the sequencing results were run through the NCBI BLAST program (https://www.ncbi.nlm.nih.gov/, accessed on 17 September 2021) for obtaining some annotation information. We finally identified a candidate protein coding gene for subsequent complementary validation.

### 4.10. Point-to-Point Analysis in Y2H System

Based on the annotation information in the database, we preliminarily identified 4 candidate interacting protein coding genes that we had predicted and one screened from the cDNA library. The CDS of these candidate genes was isolated from the total cDNA of the Chinese Spring wheat cultivar. These genes were recombined into pGADT7 vector to construct the prey vector; pGBKT7-N+domain was used as bait, pGBKT7-53+ pGADT7-T was used as positive control and pGBKT7-Lam + pGADT7-T as negative control, and recombinant bait vectors and prey vector were respectively combined and co-transformed into yeast strain Y2H, then cultured on SD/-Trp/-Leu solid medium for 3 days. Then, the detected positive yeast monoclonal plaque was transferred to SD/-Trp/-Leu/-His/-Ade/+X-α-Gal solid medium for further culture for about 3 days, and we observed whether yeast could grow normally and show blue color.

### 4.11. Bimolecular Fluorescence Complementation (BiFC) Analysis

First, the recombinant vectors NeYFP-*TaTOE1-B1-3*, CeYFP-*ARF16-B1*, and CeYFP-*TaPIFI* were constructed, and the recombinant plasmid with correct sequencing was transferred into *Agrobacterium tumefaciens* GV3101 (P-SOUP) strain. Positive clones were selected and propagated in liquid LB (50 μg/mL Kan, 25 μg/mL Rif, and 50 μg/mL Gen). The bacteria were suspended by using the resuspension solution described above, and the OD_600_ was adjusted to 0.8. The resuspended NeYFP-*TaTOE1-B1-3* strain was mixed with CeYFP-*TaPIFI* or CeYFP-*ARF18* at a volume ratio of 1:1. After being placed in the dark for 1–3 h, the tobacco leaves were injected with syringes. Two days later, fluorescence signals were observed by using a confocal microscope.

## Data Availability

The data presented in this study are available in the article and Supplementary Materials.

## Supplementary Materials

The following are available online at https://www.mdpi.com/article/10.3390/ijms222312645/s1.

## References

[1] Niu X.M., Xu Y.C., Li Z.W., Bian Y.T., Hou X.H., Chen J.F., Zou Y.P., Jiang J., Wu X., Ge S. (2019). Transposable elements drive rapid phenotypic variation in Capsella rubella. Proc. Natl. Acad. Sci. USA.

[2] Zhao X.B., Fu X.D., Yin C.B., Lu F. (2021). Wheat speciation and adaptation: Perspectives from reticulate evolution. aBIOTECH.

[3] Laurent C., George C. (2006). The quest for florigen: A review of recent progress. J. Exp. Bot..

[4] Shaw L.M., Turner A.S., Laurie D.A. (2012). The impact of photoperiod insensitive *Ppd-D1a* mutations on the photoperiod pathway across the three genomes of hexaploid wheat (*Triticum aestivum*). Plant J..

[5] Xu S.J., Chong K. (2018). Remembering winter through vernalization. Nat. Plants.

[6] Bao S.J., Hua C.M., Shen L.S., Yu H. (2020). New insights into gibberellin signaling in regulating flowering in *Arabidopsis*. J. Integr. Plant Biol..

[7] Afzal F., Li H., Gul A., Subhani A., Ali A., Mujeeb K.A., Rasheed A. (2019). Genome-Wide analyses reveal footprints of divergent selection and drought adaptive traits in synthetic-derived wheats. G3 Genes Genomes Genet..

[8] Abdennour S., Houcine B., Rhouma S., Sahbi F., Tahar S. (2019). Stability and adaptability concepts of bread wheat (*Triticum aestivum* L.) in the northwest of Tunisia. Biol. Futura.

[9] Akram S., Arif M.A.R., Hameed A. (2021). A GBS-based GWAS analysis of adaptability and yield traits in bread wheat (*Triticum aestivum* L.). J. Appl. Genet..

[10] Worland A.J. (1996). The influence of flowering time genes on environmental adaptability in European wheats. Euphytica.

[11] Kamran A., Iqbal M., Spaner D. (2014). Flowering time in wheat (*Triticum aestivum* L.): A key factor for global adaptability. Euphytica.

[12] Streck N.A., Weiss A., Baenziger P.S. (2003). A generalized vernalization response function for winter wheat. Agron. J..

[13] Yan L.L., Loukoianov A., Tranquilli G., Helguera M., Fahima T., Dubcovsky J. (2003). Positional cloning of the wheat vernalization gene *VRN1*. Proc. Natl. Acad. Sci. USA.

[14] Yan L.L., Loukoianov A., Blechl A., Tranquilli G., Ramakrishna W., SanMiguel P., Dubcovsky J. (2004). The wheat *VRN2* gene is a flowering repressor down-Regulated by vernalization. Science.

[15] Li G.Q., Yu M., Fang T.L., Cao S.H., Brett F.C., Yan L.L. (2013). Vernalization requirement duration in winter wheat is controlled by *TaVRN-A1* at the protein level. Plant J..

[16] Kippes N., Debernardi J.M., Vasquez-Gross H.A., Akpinar B.A., Budak H., Kato K., Dubcovsky J. (2015). Identification of the *VERNALIZATION 4* gene reveals the origin of spring growth habit in ancient wheats from South Asia. Proc. Natl. Acad. Sci. USA.

[17] Zhao Y.Y., Wang X., Wei L., Wang J.X., Yin J. (2016). Characterization of *Ppd-D1* alleles on the developmental traits and rhythmic expression of photoperiod genes in common wheat. J. Integr. Agric..

[18] Errum A., Rehman N., Khan M.R., Ali G.M. (2021). Genome-wide characterization and expression analysis of pseudo-response regulator gene family in wheat. Mol. Biol. Rep..

[19] Zhang B.L., Wang L., Zeng L.P., Zhang C., Ma H. (2015). *Arabidopsis* TOE proteins convey a photoperiodic signal to antagonize CONSTANS and regulate flowering time. Genes Dev..

[20] Okamuro J.K., Caster B., Villarroel R., Van Montagu M., Jofuku K.D. (1997). The AP2 domain of APETALA2 defines a large new family of DNA binding proteins in *Arabidopsis*. Proc. Natl. Acad. Sci. USA.

[21] Cui L.C., Feng K.W., Wang M.X., Wang M., Deng P.C., Song W.N., Nie X.J. (2016). Genome-Wide identification, phylogeny and expression analysis of AP2/ERF transcription factors family in *Brachypodium distachyon*. BMC Genom..

[22] Simons J.K. (2005). Molecular characterization of the major wheat domestication gene *Q*. Genetics.

[23] Greenwood J.R., Finnegan E.J., Watanabe N., Trevaskis B., Swain S.M. (2017). New alleles of the wheat domestication gene *Q* reveal multiple roles in growth and reproductive development. Development.

[24] Liu P., Liu J., Dong H.X., Sun J.Q. (2017). Functional regulation of *Q* by microRNA172 and transcriptional co-repressor TOPLESS in controlling bread wheat spikelet density. Plant Biotechnol. J..

[25] Xu B.J., Chen Q., Zheng T., Jiang Y.F., Qiao Y.Y., Guo Z.R., Qi P.F. (2018). An overexpressed *Q* allele leads to increased spike density and improved processing qualityin common wheat (*Triticum aestivum*). G3 Genes Genomes Genet..

[26] Elliott R.C., Betzner A.S., Huttner E., Oakes M.P., Tucker W.Q., Gerentes D., Smyth D.R. (1996). *AINTEGUMENTA*, an APETALA2-like gene of *Arabidopsis* with pleiotropic roles in ovule development and floral organ growth. Plant Cell.

[27] Zhao L.F., Xu S.B., Chai T.Y., Wang T. (2006). *OsAP2-1*, an AP2-like gene from *Oryza sativa*, is required for flower development and male fertility. Sex Plant Reprod..

[28] Krizek B. (2009). *AINTEGUMENTA* and *AINTEGUMENTA-LIKE6* act redundantly to regulate *Arabidopsis* floral growth and patterning. Plant Physiol..

[29] Moose S.P., Sisco P.H. (1996). *Glossy15*, an APETALA2-like gene from maize that regulates leaf epidermal cell identity. Genes Dev..

[30] Zhu Q.H., Helliwell C.A. (2011). Regulation of flowering time and floral patterning by miR172. J. Exp. Bot..

[31] Zikhali M., Wingen L.U., Leverington-Waite M., Specel S., Griffiths S. (2017). The identification of new candidate genes *Triticum aestivum FLOWERING LOCUS T3-B1* (*TaFT3-B1*) and *TARGET OF EAT1* (*TaTOE1-B1*) controlling the short-day photoperiod response in bread wheat. Plant Cell Environ..

[32] Jung J.H., Seo Y.H., Seo P.J., Jose L.R., Yun J., Chua N.H., Park C.M. (2007). The GIGANTEA-Regulated MicroRNA172 mediates photoperiodic flowering independent of *CONSTANS* in *Arabidopsis*. Plant Cell.

[33] Jung J.H., Lee S.M., Yun J., Lee M.Y., Park C.M. (2014). The miR172 target *TOE3* represses *AGAMOUS* expression during *Arabidopsis* floral patterning. Plant Sci..

[34] Kang G.Z., Xu W., Liu G.Q., Peng X.Q., Guo T.C. (2013). Comprehensive analysis of the transcription of starch synthesis genes and the transcription factor *RSR1* in wheat (*Triticum aestivum*) endosperm. Genome.

[35] Wang M.J., Ding L., Liu X.H., Liu J.X. (2021). Two B-box domain proteins, BBX28 and BBX29, regulate flowering time at low ambient temperature in *Arabidopsis*. Plant Mol. Biol..

[36] Prem N. (2014). Identifying Objectives for breeding improved vegetable varieties-hard but vital Choice. J. Food Nutr. Res..

[37] Syed N.H., Kalyna M., Marquez Y., Barta A., Brown J.W. (2012). Alternative splicing in plants-coming of age. Trends Plant Sci..

[38] Swarup R., Crespi M., Bennett M.J. (2016). One Gene, Many Proteins: Mapping cell-specific alternative splicing in plants. Dev. Cell.

[39] Jiang G.X., Zhang D.D., Li Z.W., Liang H.L., Deng R.F., Su X.G., Jiang Y.M., Duan X.W. (2021). Alternative splicing of *MaMYB16L* regulates starch degradation in banana fruit during ripening. J. Integr. Plant Biol..

[40] Simpson C.G., Hedley P.E., Watters J.A., Clark G.P., McQuade C., Machray G.C., Brown J.W. (2000). Requirements for mini-exon inclusion in potato invertase mRNAs provides evidence for exon-scanning interactions in plants. Cold Spring Harb. Lab. Press.

[41] Zabala G., Vodkin L. (2007). Novel exon combinations generated by alternative splicing of gene fragments mobilized by a CACTA transposon in *Glycine max*. BMC Plant Biol..

[42] Qüesta J.I., Song J., Geraldo N., An H., Dean C. (2016). *Arabidopsis* transcriptional repressor VAL1 triggers Polycomb silencing at FLC during vernalization. Science.

[43] Shim J.S., Kubota A., Imaizumi T. (2017). Circadian clock and photoperiodic flowering in *Arabidopsis*: CONSTANS is a hub for signal integration. Plant Physiol..

[44] Hwang K., Susila H., Nasim Z., Jung J.Y., Ahn J.H. (2019). *Arabidopsis* ABF3 and ABF4 transcription factors act with the NF-YC complex to regulate *SOC1* expression and mediate drought-accelerated flowering. Mol. Plant.

[45] Wang D., Portis A.R. (2007). A novel nucleus-encoded Chloroplast protein, PIFI, is involved in NAD(P)H dehydrogenase complex-mediated Chlororespiratory Electron Transport in *Arabidopsis*. Plant Physiol..

[46] Dal Bosco C., Lezhneva L., Biehl A., Leister D., Strotmann H., Wanner G., Meurer J. (2004). Inactivation of the Chloroplast ATP synthase γ subunit results in high non-photochemical fluorescence quenching and altered nuclear gene expression in *Arabidopsis thaliana*. J. Biol. Chem..

[47] Zhang X., Henriques R., Lin S.S. (2006). *Agrobacterium*-mediated transformation of *Arabidopsis thaliana* using the floral dip method. Nat. Protocols.

[48] Tomasz C., Mark S., Thomas A., Michael K.U., Wolf-Rüd S. (2005). Genome-Wide identification and testing of superior reference genes for transcript normalization in *Arabidopsis*. Plant Physiol..

[49] Li Z., Ma S.C., Liu D., Zhang L.L., Du X.J., Xia Y., Song Q.L., Li Y., Zhang Y.M., Li Z.L. (2020). Morphological and proteomic analysis of young spikes reveals new insights into the formation of multiple-pistil wheat. Plant Sci..

[50] Wei F., Song T.Q., Zhou J.F., Cheng J., Li R.B., Yu M., Zhang Y.R., Yu Y., Zhang B., Zhang X.K. (2021). A transcription factor TaTCP20 regulates the expression of *Ppd-D1b* in common wheat. Plant Biotechnol. Rep..

