# Supplementary material for: A Wheat TaTOE1-B1 Transcript TaTOE1-B1-3 Can Delay the Flowering Time of Transgenic Arabidopsis
Source: Int J Mol Sci. 2021 Nov 23;22(23):12645. doi: 10.3390/ijms222312645 (PMC8657464; doi:10.3390/ijms222312645)
Supplement: Supplementary file 1 [file ijms-22-12645-s001.zip › ijms-1448711-supplementary.pdf]

Supplementary material

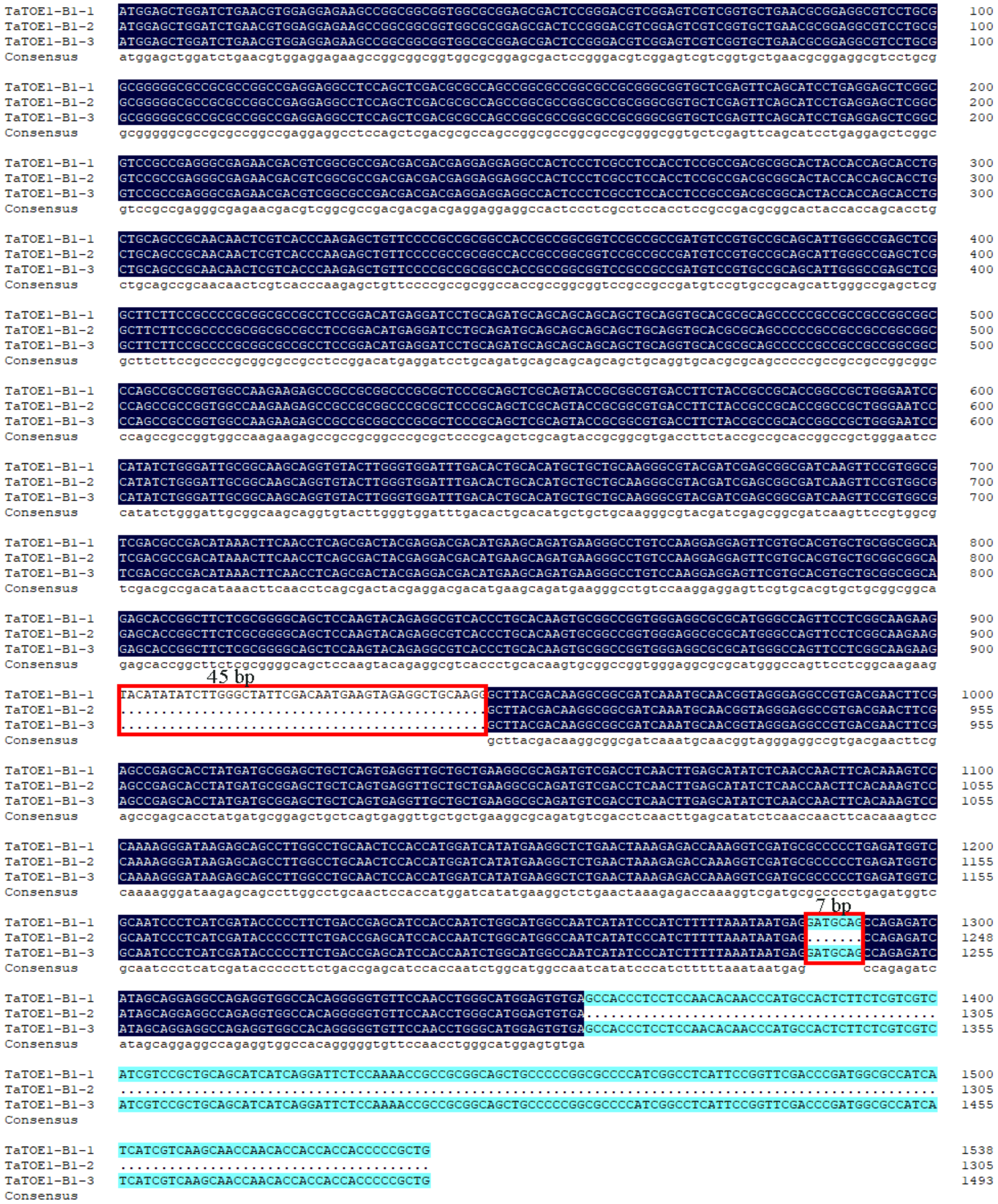

Figure S1. Coding Sequence alignment of the three transcripts produced by *TaTOE1-B1* in this syudy. *TaTOE1-B1-1* (ID: TraesCS1B02G076300.1), *TaTOE-B1-2* (ID: TraesCS1B02G076300.2) and *TaTOE1-B1-3* (ID: TraesCS1B02G076300.3); The red boxes show the locations of the 45bp (1219-1264) and 7bp (1603-1611) differential loci, respectively.

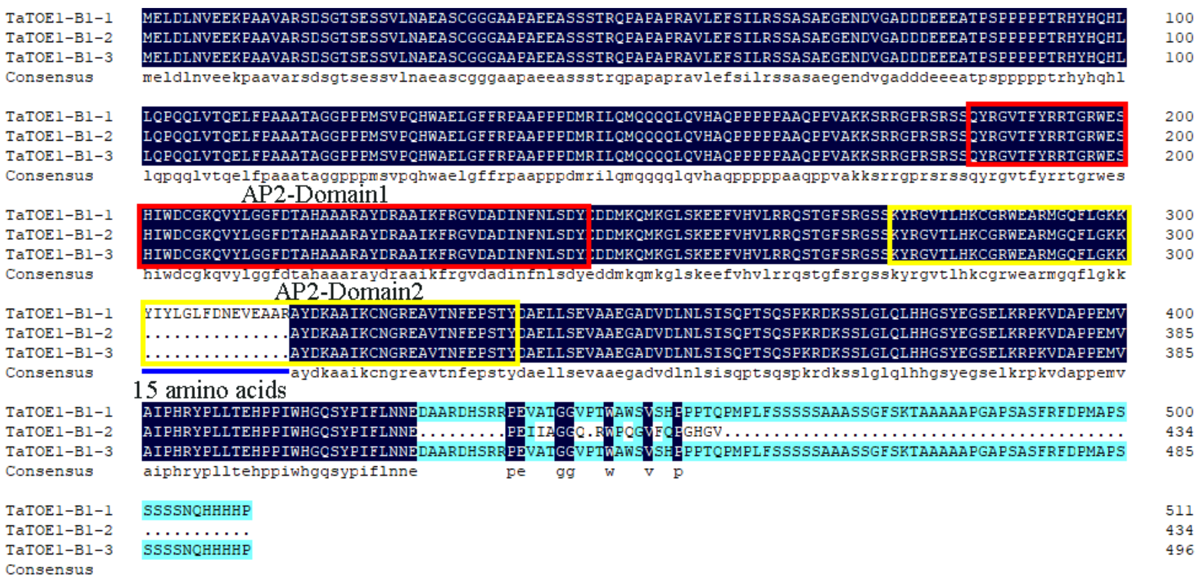

Figure S2. Protein sequence alignment of TaTOE1-B1-1, TaTOE-B1-2 and TaTOE1-B1-3. The red box represents the first AP2 domain (185-246), and the yellow box represents the second (277-338); The blue line shows 15 different amino acid locations.

Table S1. Primers used in this study

Primer name	Sequences (5'-3')
Clone-F	ATGGAGCTGGATCTGAACGTGGAGGAG
Clone-R	TTTTCAGCGGGGGTGGTGGTGGTG
pBI-CDs-F	acgggggactctagaggatccATGGAGCTGGATCTGAACGTGG
pBI-CDs-R	cgatcggggaaattcgagctcTCAGCGGGGGTGGTGGTG
FT-F	CTGGAACAACCTTTGGCAAT
FT-R	AGCCACTCTCCCTCTGACAA
CO-F	ATTCTGCAAACCCACTTGCT
CO-R	CCTCCTTGGCATCCTTATCA
FLC-F	AGCCAAGAAGACCGAACTCA
FLC-R	GGAGATTTGTCCAGCAGGTG
SOC1-F	AATTCGCCAGCTCCAATATG
SOC1-R	CCTCGATTGAGCATGTTCCTA
PP2A-F	TAACGTGGCCAAAATGATGC
PP2A-R	GTTCTCCACAACCGCTTGGT
Sub-F	atctcgagctcaagcttcgaaATGGAGCTGGATCTGAACGTGG
Sub-R	ccgtcgactgcagaattcgaaGCGGGGGTGGTGGTGGTG
PGBKT7-F	atggccatggaggccgaattcATGGAGCTGGATCTGAACGTGG
PGBKT7-R	atgcggccgctgcaggtcgacTCAGCGGGGGTGGTGGTG
N-R	atgcggccgctgcaggtcgacCGAGCTGCGGGAGCG
N+Domain-R	atgcggccgctgcaggtcgacATCATAGGTGCTCGGCTC
C-F	atggccatggaggccgaattcATGGCGGAGCTGCTCAATG
AD-TaTCP5-A1-F	gccatggaggccagtgaattcATGATAAGCGCCGGCGAC
AD-TaTCP5-A1-R	cagctcgagctcgatggatccTCATCGATCAACCCTGGCTT
AD-TaARF16-B1-F	gccatggaggccagtgaattcATGAAGGAGGTGGGCGAGG
AD-TaARF16-B1-R	cagctcgagctcgatggatccTCACACCCGCTGAACCATG
AD-TaSBP1-D6-F	gccatggaggccagtgaattcATGGCTGTGCAGGCCCAG
AD-TaSBP1-D6-R	cagctcgagctcgatggatccTCAACACAGGATGGCCTCCA
AD-TaMYB85-D5-F	gccatggaggccagtgaattcATGGGGCGGCAGCCGTGC
AD-TaMYB85-D5-R	cagctcgagctcgatggatccCTAGAGATTTGATCCACTTGAGCTGT
TaTOE1-B1-3-NYFP-F	atctcgagctcaagcttcgaaATGGAGCTGGATCTGAACGTGG
TaTOE1-B1-3-NYFP-R	ccgtcgactgcagaattcgaaGCGGGGGTGGTGGTGGTG
TaARF16-B1-CYFP-F	atctcgagctcaagcttcgaaATGAAGGAGGTGGGCGAGG
TaARF16-B1-CYFP-R	ccgtcgactgcagaattcgaaTCACACCCGCTGAACCATG
TaPIFI-CYFP-F	atctcgagctcaagcttcgaaATGGCCACCATCTCTTCTTGC
TaPIFI-CYFP-R	ccgtcgactgcagaattcgaaCTCCTCCGTATCAGAGTCCAGC

The lowercase letters indicate homologous arm sequences, and the underscores indicate the restriction site sequence.
